# Isavuconazole Population Pharmacokinetic Analysis Using Nonparametric Estimation in Patients with Invasive Fungal Disease (Results from the VITAL Study)

**DOI:** 10.1128/AAC.00514-16

**Published:** 2016-07-22

**Authors:** Laura L. Kovanda, Amit V. Desai, Qiaoyang Lu, Robert W. Townsend, Shahzad Akhtar, Peter Bonate, William W. Hope

**Affiliations:** a Antimicrobial Pharmacodynamics and Therapeutics, Department of Molecular and Clinical Pharmacology, Institute of Translational Medicine, University of Liverpool, Liverpool, United Kingdom; b Astellas Pharma Global Development, Inc., Northbrook, Illinois, USA

## Abstract

Isavuconazonium sulfate (Cresemba; Astellas Pharma Inc.), a water-soluble prodrug of the triazole antifungal agent isavuconazole, is available for the treatment of invasive aspergillosis (IA) and invasive mucormycosis. A population pharmacokinetic (PPK) model was constructed using nonparametric estimation to compare the pharmacokinetic (PK) behaviors of isavuconazole in patients treated in the phase 3 VITAL open-label clinical trial, which evaluated the efficacy and safety of the drug for treatment of renally impaired IA patients and patients with invasive fungal disease (IFD) caused by emerging molds, yeasts, and dimorphic fungi. Covariates examined were body mass index (BMI), weight, race, impact of estimated glomerular filtration rate (eGFR) on clearance (CL), and impact of weight on volume. PK parameters were compared based on IFD type and other patient characteristics. Simulations were performed to describe the MICs covered by the clinical dosing regimen. Concentrations (*n* = 458) from 136 patients were used to construct a 2-compartment model (first-order absorption compartment and central compartment). Weight-related covariates affected clearance, but eGFR did not. PK parameters and intersubject variability of CL were similar across different IFD groups and populations. Target attainment analyses demonstrated that the clinical dosing regimen would be sufficient for total drug area under the concentration-time curve (AUC)/MIC targets ranging from 50.5 for Aspergillus spp. (up to the CLSI MIC of 0.5 mg/liter) to 270 and 5,053 for Candida albicans (up to MICs of 0.125 and 0.004 mg/liter, respectively) and 312 for non-albicans Candida spp. (up to a MIC of 0.125 mg/liter). The estimations for Candida spp. were exploratory considering that no patients with Candida infections were included in the current analyses. (The VITAL trial is registered at ClinicalTrials.gov under number NCT00634049.)

## INTRODUCTION

Isavuconazonium sulfate is the water-soluble prodrug of the novel, broad-spectrum, triazole antifungal agent isavuconazole and is available in cyclodextrin-free intravenous (i.v.) and oral (p.o.) formulations (1). The U.S. Food and Drug Administration and the European Medicines Agency recently approved isavuconazole for the treatment of invasive aspergillosis (IA) and invasive mucormycosis (IM). The approval in the European Union for mucormycosis is for patients for whom amphotericin B is inappropriate. Many of the pharmacokinetic (PK) analyses reported to date are limited to healthy volunteers and patients treated for IA. There is a paucity of data regarding the population pharmacokinetics (PPK) in patients with a variety of invasive fungal infections and underlying diseases.

*In vitro* data for isavuconazole have demonstrated broad-spectrum activity against a variety of fungi, including Aspergillus spp., Candida spp., Mucorales, Cryptococcus spp., and dimorphic and other rare fungi (2). *In vivo* models have also been conducted, demonstrating concentration-dependent antifungal activity for the treatment of invasive pulmonary aspergillosis, disseminated aspergillosis, disseminated candidiasis, mucormycosis, and cryptococcal meningitis (3–7). The VITAL clinical trial evaluated the efficacy and safety of isavuconazole for treatment of a variety of invasive fungal diseases (IFD), including the treatment of IA in patients with renal impairment and treatment of patients with diseases caused by emerging molds, yeasts, and dimorphic fungi. Isavuconazole demonstrated successful outcomes for patients with IA and IM, with all-cause mortality rates through day 42 of 12.5% and 37.8%, respectively, for these patients. Successful outcomes have also been reported for patients with IA and renal impairment and for patients with infections caused by Cryptococcus spp., dimorphic fungi (such as Coccidioides spp. and Paracoccidioides spp.), Fusarium spp., and Scedosporium spp. (8–15).

A previous PPK analysis was conducted using isavuconazole concentration-time data collected from nine phase 1 PK studies of healthy subjects and the phase 3 SECURE trial of patients with IA (16). These analyses demonstrated that a 3-compartment model fit the data well (absorption, central, and peripheral compartments). The PK profiles were similar between healthy subjects and patients with IA. In addition, isavuconazole demonstrated linear PK over the range of dosages (40 to 400 mg) that were examined.

An understanding of the PK of novel compounds in various patient populations is a first critical step for optimal clinical use. The phase 3 VITAL trial provides the opportunity to compare PK characteristics for a population of patients infected with a variety of fungal pathogens. This is especially important for critically ill or severely immunosuppressed patients and in circumstances where organ function is compromised. In the current analysis, we constructed a PPK model for patients enrolled in the phase 3 VITAL trial, with the goal of comparing PK across the different patient populations to further understand the optimal use of isavuconazole.

## MATERIALS AND METHODS

### Study design.

The VITAL trial (ClinicalTrials.gov registration number NCT00634049) evaluated the efficacy and safety of isavuconazole for the primary treatment of proven or probable IA in patients with renal impairment and for treatment of patients with proven or probable IFD caused by Mucorales and other emerging molds, yeasts, and dimorphic fungi. Patients were eligible whether they required primary therapy or were refractory or intolerant to their previous systemic antifungal therapy. Patients received a loading regimen of either i.v. or p.o. isavuconazonium sulfate at a dose of 372 mg (equivalent to 200 mg isavuconazole) every 8 h for the first 48 h, followed by i.v. or p.o. isavuconazonium sulfate at 372 mg once daily for up to a maximum of 180 days. The institutional review board at each center approved the study protocol, and all research subjects provided written informed consent.

### Plasma PK sampling.

Blood samples were collected from each patient on treatment days 7, 14, and 42 and at the end of therapy. Collection was targeted at the time point 24 h after the start of the infusion or the p.o. dose the previous day (i.e., trough concentration). Full 24-h profiles were obtained for a subset of 33 patients. After collection, samples were processed immediately and stored at −80°C until shipment to the central research laboratory.

### Bioanalytical analysis.

Isavuconazole (BAL4815) and inactive cleavage product (BAL8728) concentrations in plasma samples were measured using a validated liquid chromatography-tandem mass spectrometry (LC-MS/MS) method at Pharmaceutical Product Development, LLC (Middleton, WI). A plasma sample volume of 50 μl was combined with the internal standard (d_4_-isavuconazole/pyridooxazinone) and subjected to protein precipitation using acetonitrile. Following centrifugation for 10 min at 5°C, 75 μl of the supernatant was isolated for dilution with water:formic acid:ammonium hydroxide (1,000:10:0.5 [vol/vol/vol])-acetonitrile (850:150 [vol/vol]) and then submitted for analysis. Chromatographic separation was achieved using a Synergi Polar-RP column (20 mm by 2.0 mm by 4 μm; Phenomenex, Torrance, CA) with a gradient mobile phase consisting of water, acetonitrile, methanol, and formic acid. An API3000 mass spectrometer (AB Sciex, Framingham, MA) in positive ion mode was used to monitor the analytes. Multiple-reaction monitoring transitions were *m/z* 438.1 → 224.1 for isavuconazole, 165.0 → 121.2 for the inactive cleavage product, 442.1 → 224.1 for d_4_-isavuconazole, and 151.1 → 123.2 for pyridooxazinone. The validated curve range was 5 to 1,250 ng/ml for both isavuconazole and the inactive cleavage product, and any samples measuring above the upper limit were diluted 5-fold, 10-fold, or 20-fold prior to analysis. The lower limit of quantification (LOQ) was 5 ng/ml. Interassay precisions (coefficients of variation [%]) of the BAL4815 quality control samples (12.5, 25, 75, 250, and 1,000 ng/ml) were 6.1%, 4.6%, 3.1%, 2.5%, and 2.6%, respectively, while the interassay accuracies (relative errors [%]) were 2.4%, 3.6%, 3.1%, 2.3%, and 0.2%, respectively. Interassay precisions of the BAL8728 quality control samples (12.5, 25, 75, 250, and 1,000 ng/ml) were 7.0%, 7.1%, 5.9%, 8.5%, and 6.5%, respectively, while the interassay accuracies were −1.9%, −0.4%, −1.0%, 2.1%, and 2.8%, respectively. All study samples were analyzed within the established long-term stability of the drug (isavuconazole) (955 days at −70°C). All PPK analyses were conducted on the isavuconazole concentrations due to the lack of antifungal activity of the cleavage product.

### PPK modeling.

A PPK model was developed using nonparametric estimation in Pmetrics software (v1.3.2; University of Southern California, Los Angeles, CA) (17). The model-fitting process included evaluations of both 2- and 3-compartment models, including absorptive compartments with and without a lag time. Data were weighted by the inverse of the estimated assay variance. Acceptance of the final model was evaluated by visual inspection of the observed versus predicted concentration values before and after the Bayesian step, the coefficient of determination (*r^2^*) from the linear regression of the observed versus predicted values, and estimates for bias (mean weighted error) and precision (adjusted mean weighted squared error).

The impacts of body mass index (BMI), weight, and race (Asian versus other) on clearance and of weight on volume were initially assessed as covariates by visual inspection of the graphical representation of each covariate versus clearance and volume to evaluate for inclusion in the final model. The covariates were chosen based on results of previous PPK studies with isavuconazole (16). In addition, this study permitted the enrollment of patients with renal impairment, in contrast to the phase 3 SECURE trial. Therefore, the impact of the estimated glomerular filtration rate (eGFR) on clearance was also evaluated. The eGFR was calculated using the serum creatinine level and the “modification of diet in renal disease” formula.

The average area under the concentration-time curve (AUC_avg_) for each patient was calculated using the Bayesian posterior parameter estimates from the final model by using the trapezoidal rule in Pmetrics. AUC_avg_ was calculated by determining the total AUC over the entire dosing period and dividing it by the number of days of therapy for each patient. The patients were subclassified according to the type of IFD diagnosed at baseline and baseline underlying disease. The PK parameters for the patient subsets were evaluated separately and compared with the overall data set and with those from the previously reported PPK model with isavuconazole (16). Statistical comparisons were performed in MYSTAT 12 (version 12.02; Systat Software, Inc.).

Simulations of 5,000 patients were performed in Pmetrics to assess the probability of target attainment (PTA) for the approved clinical dosing regimen to achieve the various total drug AUC/MIC pharmacodynamic (PD) targets estimated from preclinical models for different organisms (i.e., Aspergillus fumigatus, Candida albicans, Candida glabrata, and Candida tropicalis) (5, 18, 19). The choice to use total drug AUC/MIC targets was considered acceptable because protein binding values are similar between humans and the animal models used to estimate the targets.

## RESULTS

In total, 458 isavuconazole concentrations from 136 patients were included in the model. Table 1 summarizes the key patient demographics and clinical characteristics. The majority of the patients were white. The mean (± standard deviation [SD]) weight and BMI were 70.2 ± 19.5 kg and 24.4 ± 5.9 kg/m^2^, respectively. Fifty-three patients (39%) had an eGFR of <60 ml/min/1.73 m^2^ at baseline, and the mean eGFR for the total population was 80.2 ml/min/1.73 m^2^. A total of 65 patients (48%) had one or more of the following: hematologic malignancy, active malignancy, or baseline neutropenia. Forty-one percent (*n* = 56) of the overall population had hematologic malignancies, and 30% (*n* = 41) had active malignancy at enrollment. One-fourth (*n* = 34) of patients were neutropenic (absolute neutrophil count [ANC] values of <500/mm^3^) at baseline. Twenty-four of the 65 patients with hematologic malignancies had all three baseline characteristics combined, 13 patients were described as having active hematologic malignancies at baseline, and 5 had both hematologic malignancies and baseline neutropenia. Fourteen were reported as having only hematologic malignancies, 4 had only active malignancies, and 5 had only neutropenia. Seventy-one patients did not have any of the three characteristics reported at baseline. Further, 17% of patients had received an allogeneic hematopoietic stem cell transplant, and the use of T-cell immunosuppressants and corticosteroids was reported for 41% and 24% of the overall population, respectively. In addition to IM (*n* = 34) and IA (*n* = 20), the analysis included patients with IFD caused by other fungal pathogens, as summarized in Table 1. After IM, the next most commonly enrolled IFD were caused by dimorphic fungi, such as Coccidioides spp., Paracoccidioides spp., and Histoplasma spp.

**TABLE 1:** VITAL patient demographics and clinical characteristics

Patient population (*n*)	No. of patients				Body wt (kg)^*a*^	BMI (kg/m^2^)^*a*^
	White	Asian	Black	Other		
Overall (136)	100	23	9	4	70.2 ± 19.5	24.4 ± 5.9
Patients with infection						
IA (20)	18	2	0	0	68.0 ± 20.7	23.2 ± 4.7
IM (34)	23	8	3	0	73.4 ± 18.6	25.3 ± 5.5
Other filamentous fungi and molds (23)	17	4	2	0	72.8 ± 25.0	25.1 ± 7.1
Dimorphic fungi (29)	20	4	2	3	64.2 ± 16.1	23.0 ± 5.8
Non-Candida yeasts (10)	5	3	1	1	61.9 ± 11.7	22.2 ± 3.3
Mixed infection (13)	11	1	1	0	75.0 ± 18.7	26.1 ± 7.5
Patients with no pathogen identified (7)	6	1	0	0	79.2 ± 20.4	27.7 ± 5.6

a Data are means ± SD.

**FIG 1:**
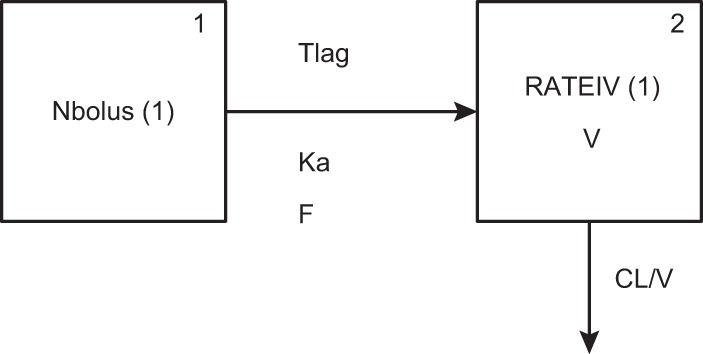
Illustration of the structural model, where compartment 1 represents the gut for oral administration and compartment 2 represents the central compartment. CL, clearance; F, bioavailability; Ka, first-order absorption rate constant; Tlag, lag time; V, volume in the central compartment.

**FIG 2:**
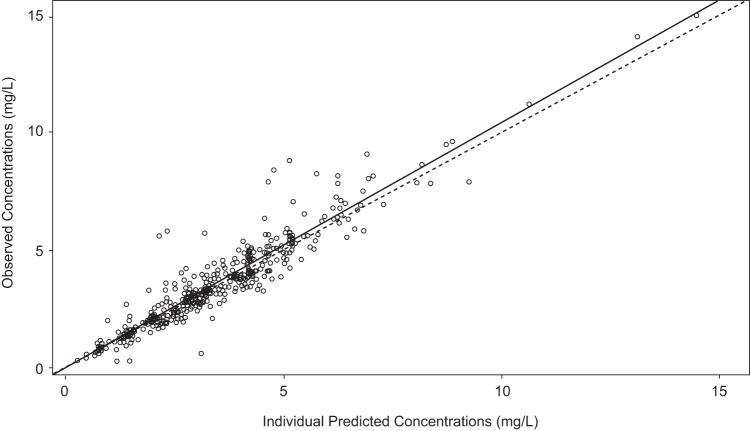
Observed versus posterior predicted concentrations (milligrams per liter) from the final model after the Bayesian step (*r^2^* = 0.89; slope = 1.05 [95% CI, 1.01 to 1.08]; intercept = −0.0484 [95% CI, −0.179 to 0.0825]). The dotted line is the line of unity where observed concentrations equal predicted concentrations.

A 2-compartment model with first-order absorption fit the data well. Figure 1 shows a schematic of the structural model. Compartment 1 represents the absorptive compartment (i.e., gut), and compartment 2 represents the central compartment. The fit of the PPK model was acceptable based on visual inspection of the observed versus predicted plots and the coefficient of determination (*r^2^*) of 0.89 for the observed versus posterior predicted values after the Bayesian step (Fig. 2). The estimates of bias and precision were also acceptable (−0.0539 and 2.77, respectively). Evaluation of a 3-compartment model did not result in a better fit of the model to the data.

**FIG 3:**
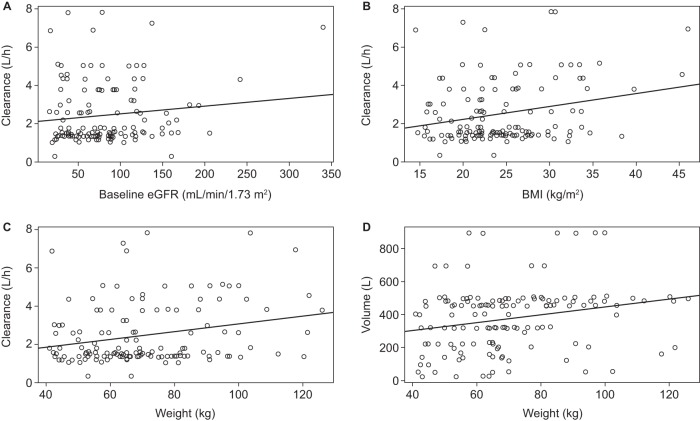
Linear regression plots charting the impacts of eGFR on clearance (A), BMI on clearance (B), weight on clearance (C), and weight on volume in the central compartment (D). (A) Linear regression for eGFR (*r^2^* and slope were 0.02 and 0 [95% CI, 0 to 0.01], respectively) versus clearance did not find a correlation. (B) There was a significant relationship between BMI and clearance (*r^2^* and slope were 0.06 and 0.07 [95% CI, 0.02 to 0.11], respectively). (C) There was a significant relationship between weight and clearance (*r^2^* and slope were 0.06 and 0.02 [95% CI, 0.01 to 0.03], respectively). (D) Linear regression showed a significant correlation between weight and volume (*r^2^* and slope were 0.06 and 2.36 [95% CI, 0.69 to 4.02], respectively).

**FIG 4:**
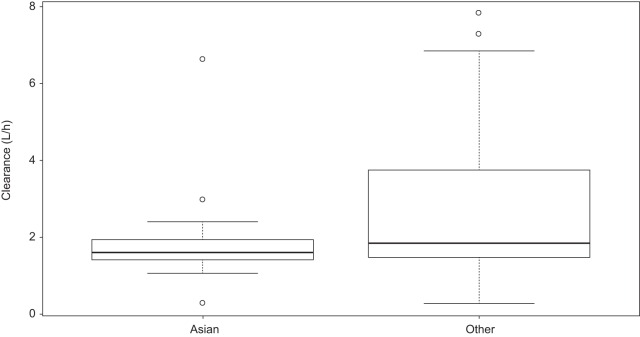
Impact of Asian race on clearance. Box plots for the clearance values for Asian patients (*n* = 23) (median = 1.59 liters/h) and other patients (i.e., non-Asian; includes white, black or African-American, and “other”; *n* = 113) (median = 1.84 liters/h) did not show a statistically significant difference (*P* = 0.06; Mann-Whitney U test).

There was not a statistically significant impact of eGFR or Asian ethnicity on clearance (Fig. 3A and 4). However, for BMI and weight, the 95% confidence interval (CI) of the slope did not include zero, suggesting statistical significance (Fig. 3B and C). Weight was found to have a significant impact on the volume of the central compartment (*r^2^* and slope were 0.06 and 2.36 [95% CI, 0.69 to 4.02], respectively) (Fig. 3D). These covariates were then incorporated into the structural model to determine if their inclusion helped to better describe interpatient variability. Only one size-related covariate (weight) was included, since other measures, such as BMI, are less practical measures in clinical settings. Inclusion of the covariates did not improve fitting, as assessed by minimal changes in −2 log likelihood (decrease of 3 points) and Akaike's information criterion (increase of 5 points) as well as by visual comparisons of the observed versus predicted concentration-time plots for each patient. Therefore, the base model was used for further evaluation of model performance.

The mean (± SD) parameter estimates for the overall population and classified according to IFD type are shown in Table 2. The mean clearance for the entire population was 2.5 ± 1.6 liters/h, and the mean AUC_avg_ was 87.1 ± 41.0 mg · h/liter. The mean values for clearance and AUC_avg_ ranged from 2.0 to 3.2 liters/h and 70.4 to 94.0 mg · h/liter, respectively, for the individual IFD types. The intersubject variability of clearance was 63% overall. AUC_avg_ and clearance values across the different types of IFD were similar (*P* = 0.420 and 0.248, respectively; Kruskal-Wallis nonparametric test). The mean estimate for bioavailability was 96.6% for the overall population (median, 98.2%).

**TABLE 2:** Isavuconazole PPK parameters in the VITAL study, based on infection^*a*^

Patient population (*n*)	CL (liters/h) (mean ± SD)	*V* (liters) (mean ± SD)	AUC_avg_ (mg · h/liter) (mean ± SD)	%CV of CL	*F* (%)
Overall (136)	2.5 ± 1.6	361.2 ± 166.3	87.1 ± 41.0	63	96.6
Patients with infection					
IA (with renal impairment) (20)	2.3 ± 1.5	412.2 ± 222.4	92.0 ± 44.0	64	97.4
IM (34)	3.0 ± 2.0	364.1 ± 186.0	87.7 ± 56.9	68	95.0
Other filamentous fungi and molds (23)	2.2 ± 0.9	344.2 ± 99.7	80.6 ± 29.2	41	96.9
Dimorphic fungi (29)	2.0 ± 1.3	343.0 ± 159.6	94.0 ± 36.7	62	97.5
Non-Candida yeasts (10)	2.5 ± 1.4	318.2 ± 160.8	92.0 ± 29.5	55	94.9
Mixed infection (13)	3.2 ± 2.0	389.8 ± 162.7	70.4 ± 24.0	62	97.7
Patients with no pathogen identified (7)	2.3 ± 1.6	340.4 ± 107.5	87.8 ± 29.1	68.7	98.2

a CL, clearance; *V*, volume in the central compartment; %CV, percent covariance of intersubject variability; *F*, bioavailability.

A further review of the PK characteristics was also explored across the different underlying diseases (Table 3). Clearance values were comparable for patients with hematologic malignancies, active malignancies, or baseline neutropenia and patients without these conditions (*P* = 0.530; Kruskal-Wallis one-way analysis of variance). The mean clearance and AUC_avg_ values for patients with hematologic malignancies were 2.8 liters/h and 85.1 mg · h/liter, respectively. Patients with a combination of hematologic malignancy, baseline neutropenia, and active malignancy also had similar values (2.8 liters/h and 85.1 mg · h/liter for clearance and AUC_avg_, respectively). Bioavailability did not vary significantly across the different underlying disease groups. The intersubject variability of clearance was consistent across the underlying disease groups and similar to the overall population value, with values ranging from 59% to 62%.

**TABLE 3:** Isavuconazole PPK parameters in the VITAL study, based on underlying disease^*a*^

Underlying disease (*n*)^*b*^	CL (liters/h) (mean ± SD)	*V* (liters) (mean ± SD)	AUC_avg_ (mg · h/liter) (mean ± SD)	%CV of CL	*F* (%)
Overall (136)	2.5 ± 1.6	361.2 ± 166.3	87.1 ± 41.0	63	96.6
Hematologic malignancy (56)	2.6 ± 1.6	378.7 ± 171.0	85.1 ± 50.2	60	96.2
Active (at baseline) malignancy (41)	2.6 ± 1.6	382.2 ± 165.3	84.4 ± 54.0	59	95.9
Baseline neutropenia (34)	2.7 ± 1.8	406.3 ± 168.0	82.8 ± 57.9	59	96.0
Combined hematologic malignancy, active (at baseline) malignancy, and baseline neutropenia (24)	2.8 ± 1.8	391.7 ± 187.2	85.1 ± 68.0	62	94.7
None^*c*^ (71)	2.8 ± 1.8	391.8 ± 187.2	85.1 ± 68.0	62	94.7

a CL, clearance; *V*, volume in the central compartment; %CV, percent covariance of intersubject variability; *F*, bioavailability.

b Patients in each category are not mutually exclusive.

c Patients without hematologic malignancy, active (at baseline) malignancy, or baseline neutropenia.

**FIG 5:**
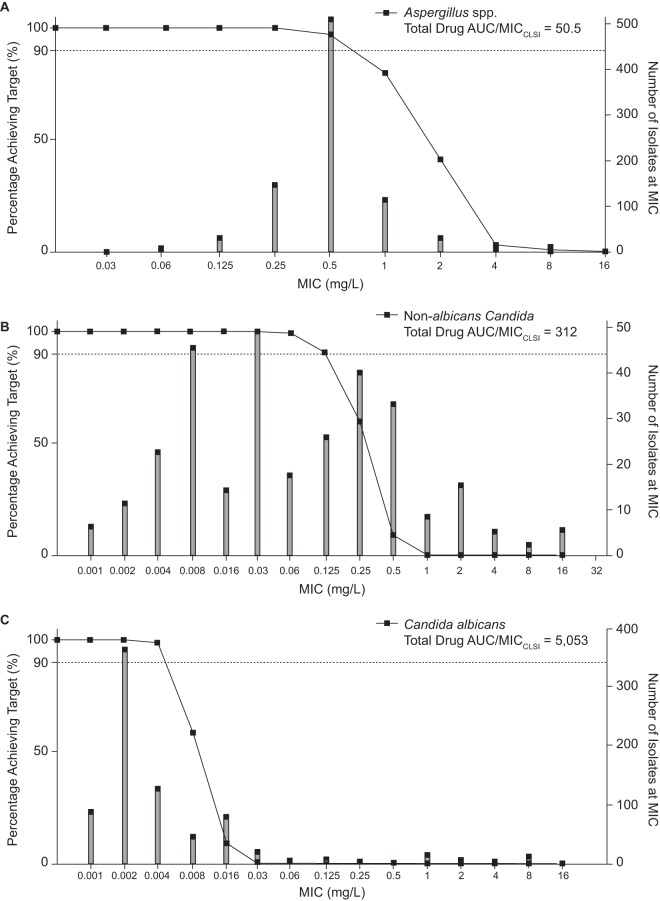
PTA (left *y* axes) for PD targets estimated in PK-PD animal models for the common fungal pathogens A. fumigatus (A), non-albicans Candida (B), and C. albicans (C). Each graph illustrates the proportion of 5,000 simulated subjects achieving each of the PD targets at each MIC value after administration of the clinical dosing regimen of isavuconazonium sulfate. Isavuconazole CLSI MIC distributions (right *y* axes) are provided for A. fumigatus (*n* = 855) (A), C. glabrata (*n* = 254) and C. tropicalis (*n* = 130) (B), and C. albicans (*n* = 844) (C).

The total drug AUC/MIC ratio is the PK-PD index that appears to best link isavuconazole exposure with efficacy in preclinical models (18, 19), and PD targets have been defined for both Aspergillus spp. and Candida spp. in a range of preclinical models (5, 18, 19). Steady-state AUCs (at day 21) for 5,000 simulated subjects were assessed over a range of MICs to determine the ability of the clinical dosing regimen to achieve the various PD targets as a function of the known MIC distribution. For Aspergillus spp., the PD target estimated by Seyedmousavi and colleagues (18) for A. fumigatus was a total drug AUC/MIC value of 50.5. For Candida spp., Lepak and colleagues (19) established targets in neutropenic mice for C. albicans (total drug AUC/MIC = 5,053) and non-albicans Candida spp. (total drug AUC/MIC = 312). Figure 5 shows the MIC coverage for each PD target for the isavuconazonium sulfate clinical dosing regimen. Depending on the species, the clinical dosing regimen would be predicted to adequately cover Clinical and Laboratory Standards Institute MIC values of 0.5 mg/liter or lower for A. fumigatus and 0.004 and 0.125 mg/liter or lower for C. albicans and non-albicans Candida spp., respectively. The same analysis was conducted using PD targets from an additional PK-PD model that was reported for isavuconazole and C. albicans (5). In this model, PD target values of 270 and 670 were estimated depending on the extent of neutropenia (temporary and persistent, respectively). If these total drug AUC/MIC targets are used for PTA analysis, the results demonstrate that the clinical dosing regimen will achieve the target for the majority of the C. albicans MIC distribution (up to MICs of 0.125 and 0.06 mg/liter, respectively), which has a MIC_90_ of 0.06 mg/liter.

## DISCUSSION

Nonparametric estimation was used to establish a PPK model for isavuconazole by using Pmetrics and plasma drug concentrations collected from phase 3 clinical trial patients being treated for a variety of IFD. A 2-compartment model including an absorptive compartment with first-order absorption fit the data well. Evaluation of the impact of covariates on clearance revealed a statistically significant relationship with BMI and weight but no relationship between either eGFR or Asian race and clearance. Weight also had a significant relationship with the volume of the central compartment. However, inclusion of the covariates in the structural model did not improve the fit of the model to the data. This is likely due to a weak relationship compared to the relatively large unexplained PK variability. The overall population parameters did not differ greatly for the individual IFD types or across the underlying diseases evaluated, even for severely immunocompromised patients.

We further assessed the adequacy of currently approved regimens by conducting PTA analyses using PD targets defined from experimental models for specific fungal pathogens. The results were consistent with previous data reported for Aspergillus spp. (16). For Candida spp., this represents the first report of a PTA analysis conducted using the PD targets for Candida spp. Importantly, however, this study did not include patients with invasive candidiasis. The majority of such cases occur in the context of critical illness, where the PK may be reasonably different and have an impact upon both the PD and PTA. Further analyses must be performed to characterize any differences in PK for patients with invasive candidiasis and then to determine if these differences have any impact on the exposure coverage of the MIC distribution for these organisms. The isavuconazole MIC_50_ and MIC_90_ for C. albicans were 0.002 and 0.06 mg/liter, respectively, for a collection of 844 isolates (20–32). For the non-albicans Candida spp. tested in the PK-PD model of disseminated candidiasis, the isavuconazole MIC_50_ and MIC_90_ were 0.03 and 1 mg/liter, respectively, for C. tropicalis (*n* = 130) and 0.06 and 1 mg/liter, respectively, for C. glabrata (*n* = 254) (20–32). If no differences in the PK of isavuconazole exist for patients with candidemia or invasive candidiasis and the patients described herein, the current analysis suggests that the clinical dosing regimen will achieve the PD target reported by Lepak and colleagues (19) for the majority of the non-albicans Candida spp. but not for C. albicans. However, a second model which evaluated the PD target for C. albicans was also explored and gave different results (5). These total drug AUC/MIC targets demonstrated that the clinical dosing regimen would achieve the target for the majority of the C. albicans MIC distribution (up to MICs of 0.125 and 0.06 mg/liter). For other important fungi, such as organisms of the Mucorales order, Cryptococcus spp., and dimorphic fungi, validated *in vivo* PK-PD models have not been fully established. This demonstrates a clear knowledge gap for optimizing therapy for life-threatening infections with these organisms.

A previous PPK model was conducted for isavuconazole by using parametric estimation in NONMEN 7.2 software (16), using plasma concentration data from nine trials involving healthy subjects and one large phase 3 clinical trial of patients with IA or infections with other filamentous fungi (SECURE trial). Desai et al. (16) employed a Weibull absorption function and did not estimate the lag time and bioavailability. In our analysis, a 2-compartment model with an absorptive compartment was better than a 3-compartment model with an absorptive compartment as previously described by Desai et al. This is likely due to sparse sampling for the large majority of patients. Regardless of the differences between the two structural models, the mean clearance values estimated from both models are similar (2.5 liters/h versus 2.4 liters/h). Important patient characteristics that potentially have an impact on drug exposure were also explored. We did not identify differences in the heterogeneous population based on IFD type or underlying diseases. The intersubject variability of clearance was consistent among the populations, even for the severely immunocompromised (i.e., patients with hematologic malignancy, active malignancy, and neutropenia combined at baseline).

We did not find a statistically significant relationship between race and clearance, in contrast to a previous report by Desai et al. that race (Asian) is associated with reduced clearance (16). The relationship between race and clearance in the current data may not have been seen because of the differences in sample size. The current analysis included 23 Asian patients, whereas the study of Desai et al. included 53 Asian patients. Desai et al. suggested that the influence of race-dependent CYPs on clearance is likely to be minimal because isavuconazole is not a substrate of CYP2D6 or CYP2C19 isoenzymes (16). However, additional pharmacogenetic studies are required to further elucidate the potential impacts of race on clearance and drug exposure. Desai et al. did not report a relationship for weight and clearance but did find a relationship between BMI and volume. Both race and weight impacts on clearance and weight impacts on volume were included in the final model. While we identified significant covariates, inclusion of the covariates in the structural model did not significantly change the model fit to the data. The reasons for this could be that the size of the Asian population and range of weights in the data set were not large enough to significantly affect the model or that the relationships of these covariates to clearance and volume are not strong.

We reported the AUC_avg_ for the population by calculating the total AUC for the dosing duration and dividing it by the number of days of therapy. It is important that the average treatment duration of therapy for the population in the PK data set was only 14 days, compared to the average duration for the study, which was 135 days. Therefore, the values for AUC_avg_ reported here are slightly lower than they would be if we were reporting steady-state AUC. The population mean estimate of AUC_avg_ was 87.1 mg · h/liter, and the mean steady-state AUC for the 5,000 simulated patients was 94.4 mg · h/liter. This was comparable to the mean estimates obtained for each of the individual IFD types and to that obtained for the patients from the SECURE trial (16).

Isavuconazole clearance and AUC were not different across the various IFD and underlying diseases from the VITAL trial and were similar to the reported clearance and AUC values for patients with IA in the SECURE analyses (16). The values for intersubject variability of clearance across the populations, even for the small group of patients with severe immunosuppression, and between the VITAL and SECURE studies were similar, further illustrating the predictability of the PK of isavuconazole in most patients. For comparison, the reported intersubject variability of AUC for posaconazole ranges from 62 to 80%, and that for voriconazole ranges from 120 to 168%, depending on the formulation administered and the patient population studied (33, 34).

PTA analysis offers an estimate of the coverage of MIC values provided by the clinical dosing regimen for common fungal pathogens. The results suggest that for the most common pathogens for which PD targets have been estimated, the clinical dosing regimen provides adequate coverage of the majority of the wild-type MIC distribution for A. fumigatus, Aspergillus flavus, and Aspergillus terreus strains, and the results support previous PTA analyses (16) for these organisms. For C. albicans and non-albicans Candida species, the results suggest reasonable coverage of the isavuconazole MIC distribution from a large collection of *in vitro* susceptibility studies. However, further analyses are necessary to characterize the PK characteristics for patients infected with invasive candidiasis and candidemia. This is especially important given that a recently completed phase 3 clinical trial of the treatment of candidemia did not meet the primary endpoint of overall response at the end of intravenous isavuconazole therapy compared to caspofungin therapy (35). If there are PK differences in critically ill patients treated for candidemia and invasive candidiasis, this may affect the outcome of the clinical breakpoint selection, and potentially the recommended dosing regimen for treatment of invasive candidiasis.
